# Action effect consistency and body ownership in the avatar-Simon task

**DOI:** 10.1371/journal.pone.0220817

**Published:** 2019-08-09

**Authors:** Christian Böffel, Jochen Müsseler

**Affiliations:** Work and Cognitive Psychology, Institute of Psychology, RWTH Aachen University, Aachen, Germany; Ghent University, BELGIUM

## Abstract

Putting oneself in the shoes of a digital alter ego becomes an increasingly relevant part of our everyday experience. In modern day psychology, these interactions can be examined within the frameworks of visual perspective taking and body ownership. Both target similar questions: What does it take, to become one with the avatar? When do we show the same behavior and make the same experiences, as if we were in its place? In this study, we want to address the role of action effect consistency for these concepts. We manipulated the participants’ sense of agency over a task-irrelevant avatar in a Simon task by providing either corresponding or random action effects. These effects could be either embodied and therefore linked to the avatar (Experiment 1) or independent of it (Experiment 2). We used stimulus-response compatibility effects from the avatar’s point of view as a measure for spontaneous visual perspective taking and a questionnaire to measure the perceived body ownership of the avatar. The results showed that corresponding action effects lead to increased spontaneous perspective taking of the avatar, regardless of whether the effect was linked to the avatar or not. Though the avatar compatibility effects were overall more pronounced in the embodied action effect condition. However, significant differences in perceived body ownership were only observed if the effects were linked to the avatar. The results might prove useful to further our understanding of subjective and objective measurements of interactions with avatars.

## Introduction

Avatars often serve as a means to interact with virtual worlds. Actions we perform with mice, keyboards or touchpads are transformed into movements of the avatar and such consequences of our actions are action effects. The anticipation of action effects plays a crucial role in guiding our actions and now represents a cornerstone of the cognitive psychology of action planning. The ideomotor principle for example [1] claims that we learn the relationship between our actions and the resulting action effects and that the subsequent anticipation of these action effects is a driving factor in action selection. If we experience, for example, that a certain switch turns the light on and if we want to illuminate the room, we can use this connection to select the appropriate action [2,3]. Even though this idea has caught a decent amount of criticism in the earlier days, (as was discussed by [4]) it has since been featured and expended on in prominent frameworks of action control such as the theory of event coding [5].

One way to examine the influence of action effects on our behavior is to apply the concept of response-effect compatibility. It has been known for quite a while that certain combinations of stimuli and responses lead to a performance advantage over others, an idea known as stimulus-response compatibility (for an overview see: [6]). A frequent observation is that tasks with a spatial overlap between stimuli and responses lead to faster responses compared to tasks without such an overlap [7]. A typical approach to study these effects is the so-called Simon task, in which the participants respond to a certain stimulus feature, such as its color, while the task-irrelevant stimulus position is varied systematically. Even though the stimulus location is task-irrelevant, the conditions with corresponding stimulus and response locations lead to a performance advantage compared to the non-corresponding conditions [8]. A similar approach can be applied to examine the relationship between responses and their action effects. In a series of experiments, Kunde [3] systematically manipulated this relationship. In the first experiment, he asked the participants to perform one of four key presses and each of the four keys was assigned a color. The task was to press the key with the same color as a stimulus presented on a computer screen. After the participants gave their responses, a white box was displayed on the screen as an action effect. In the congruent condition, the position of the action effect matched the position of the key. In the incongruent condition, the action effect was displayed at a different position instead. The results revealed a significant reaction time advantage for the congruent condition over the incongruent one. Because the action effect followed the response, it is impossible that the action effect directly affects the response, if we only look at a single isolated trial. Instead, the results are only plausible if the participants did indeed learn the action-action effect relation and therefore anticipated the action effect in later trials. These correspondence advantages in response-effect pairings are not limited to spatial effect features and can for example also be observed in intensity [9], duration [10] or valence [11].

Using spatially neutral stimuli Ansorge [12] was able to demonstrate the importance of intention in effect compatibility. The participants were asked to move a centrally presented stimulus to the left or right by pressing left or right keys and Ansorge observed a compatibility effect with faster reaction times when the direction of the movement and the position of the key corresponded. However, this was only the case, when the participants were instructed to perform this movement and therefore had the intention to move the stimulus [12].

Following the reasoning of the theory of event coding [5], action-effect integration should take place if both, the action and its effect, overlap in time [13] and it has been shown that this integration is disrupted if the delay between action and effect is larger than 1 second [14]. In general, three major conditions have been identified that benefit action-action effect anticipation: 1) A small delay between action and effect, 2) the effect only occurs rarely without the action and 3) the effect appears sufficiently often [14].

But how does action effect anticipation work, if the effect is linked to another agent? At first glance it seems difficult to anticipate an action effect that is performed by or through someone else. But we know that people interacting with avatars often show the same behavioral tendencies we would expect if they were actually in the avatar’s place [15,16]. If we present an avatar next to a vertical stimulus set in an orthogonal Simon task, we observe similar compatibility effects, as if the stimuli were regarded from the avatar’s perspective, overwriting the orthogonal Simon effect. A stimulus at the top position is compatible with a left response, when the avatar is on the left side but compatible to a right response, when the avatar is on the right. The usually observed advantage of top to right and bottom to left mappings compared to the reverse is eliminated [15]. Such avatar-based compatibility effects can be interpreted within the framework of visual perspective taking [17], but also fall in line with predictions by the referential coding account [18,19]. In the current study, we will focus on the human-like aspect of the avatar and therefore think that the visual perspective taking framework is more appropriate, especially since broader definitions of visual perspective taking are not limited to real humans or animated objects as perspective taking targets [20]. Recent studies have shown that the perspective information provided by the avatars is processed automatically, which differentiates avatars from simple objects with perspective information, such as arrows [21]. If these avatars also exhibit movements that correspond to the person’s own responses, this seems to further facilitate perspective taking of the avatar [15]. Pfister et al. [22] also investigated action effect compatibility between participants and a virtual agent and found that responses were facilitated when the virtual agent imitated the duration of a key press performed by the participants compared to conditions in which a different duration was shown as an action effect.

But the action effect itself is not the only important factor. It is also crucial how the effect is interpreted. This can have a significant influence on subjective measures of the interaction experience such as perceived body ownership of the avatar [23], the feeling that the avatar is a part of the person’s own body [24]. In a design context, for example in game design, it might be worthwhile to dilute the distinction between a person and her/his avatar in order to increase immersion [25] and create a more realistic and engaging experience.

But not only subjective measures are affected by the interpretation of action effects. A similar case can be made for objective measures of visuo-spatial perspective taking, for example measured as compatibility effects in reaction times from the avatar’s point of view. In a recent study [23], we influenced the participant’s interpretation of an action effect by giving two different explanations of the exact same action effect. The participants performed a stimulus-response compatibility task from the avatar’s point of view and the avatar always moved the hand that corresponded to the response hand. However, the interpretation of this effect was manipulated in the instruction: One group was told that the action effect stems from the avatar’s own intention to move its hands and is only indirectly influenced by the participants. The other group was instructed that they directly control the avatar akin to a tool. Even though both conditions were identical on a purely physical level, the instruction differences led to different results. We found that the direct control group showed stronger compatibility effects from the avatar’s point of view and reported higher levels of perceived body ownership of the avatar compared to the indirect control group [23]. These results are particularly interesting, because they seem to call the social aspect of visual perspective taking into question. The indirect control condition described an independent agent and was therefore closer to a real interaction scenario than the direct control condition, which resembled a tool-use scenario.

In the following experiments, we wanted to further examine the role of such action effects for perspective taking when performing a task with an avatar. In contrast to the top-down approach of [23], we aimed to employ a bottom-up approach by not explicitly stating the nature and role of the action effects. Instead, our goal was to systematically vary the correspondence between the response location and the effect location, as well as the relationship between effect and avatar.

## Experiment 1

The first experiment used avatar hand movements as action effects in a Simon task akin to the paradigm described in [15] (for a similar approach with hands instead of full avatars see [26]). The participants responded to the color of discs presented above or below the screen center with right or left key presses. Additionally, an avatar was presented either on the right or left side facing the stimuli. Based on the results of previous studies, we expected that the stimulus positions would be coded within the reference frame provided by the avatar on a horizontal axis. This was expected to cause spatial correspondence between the now left and right stimuli to the left and right responses. The resulting compatibility effects from the avatar’s point of view were used as an objective measure for visual perspective taking. Following the participant’s key press, the avatar moved either its right or left hand as an action effect and the participants performed the task in two different conditions: In the corresponding condition, the action effect was always performed by the avatar’s hand that corresponded to the participant’s response hand. Even though the connection between action and effect was not revealed in the instruction and the action effect was not defined as the action goal, we expected that the acquisition of the action-effect connection would be rather easy, because it followed the principle of anatomical correspondence and was shown instantaneously. We also predicted that this condition would lead to relatively high values of perceived body ownership of the avatar, as the avatar and the action effect would both be included in the response code. Because a predictable action effect allows the participants to reliably interact with the scenario on the screen, the action effect and therefore the avatar are valuable to evaluate a performed action or to plan a future one. In contrast to this, we also used a random action effect condition, in which the action effect did not follow anatomical correspondence but was instead random, with an expected value of 50% corresponding and non-corresponding action effects. We expected that this condition would lead to difficulties connecting action and effect and make it less likely that the effect enters the response code necessary for action planning. This should result in less perceived body ownership of the avatar. Because the avatar and the effect are not a reliable source of information for action planning, the influence of the avatar should be reduced and the compatibility effect from the avatar’s point of view diminished, indicating a decrease of spontaneous perspective taking. In Summary, we expected the following results:

Firstly, we expected to replicate the overall performance advantage of conditions that are compatible from the avatar’s point of view, with faster response times and fewer errors, compared to incompatible conditions. Secondly, we predicted that this avatar-Simon effect is larger in the corresponding action effect condition, compared to the random action effect condition. Thirdly, we expected higher reported ownership and agency values with corresponding compared to random action effects.

### Method

#### Participants

A total of 24 students (22 female) from RWTH Aachen University with a mean age of *M* = 20.9 (*SD* = 2.2) participated in this experiment for course credit. All participants reported normal or corrected-to-normal vision and were naïve to the purpose of this study.

All participants gave written and informed consent in accordance with the Declaration of Helsinki (2013) and participation was voluntary. Further, no undue physical or psychological stress by participating in this study was anticipated and the data obtained on individual participants were not used to elucidate properties of the participant but to examine general laws of cognitive information processing. Furthermore, the study did not involve deception and had no risk of physical injury. Because the study is a basic reaction time experiment, it did not pose a risk of psychological, social or economic harm to the participants. As a result, no ethical concerns were identified in accordance to the ethics guidelines of the DFG (Deutsche Forschungsgemeinschaft, [German Research Foundation], 2009 [27]) and therefore no further evaluation by an ethics committee was sought.

The sample size was determined using G*Power [28] and the predicted effect size is based on a rough estimate of effect sizes typically observed in similar paradigms. In a previous study [15, Experiment 1], we found an overall avatar compatibility effect of η_p_^2^ = .40 and a non-centrality parameter of λ = 14.8, with the same sample size of 24 participants. If we take this effect as a reference point, the current study is expected to have a power of approximately (1-β) = .96, with α = .05 with regards to the avatar-compatibility main effect in the within-subjects ANOVA. Since we had no data to estimate the size of the avatar-compatibility*action effect interaction, we decided to base our sample size on the main effect instead. However, it is likely that the avatar effect in this study could be even larger if it is enhanced by the action effect manipulation.

#### Apparatus and stimuli

**Fig 1:**
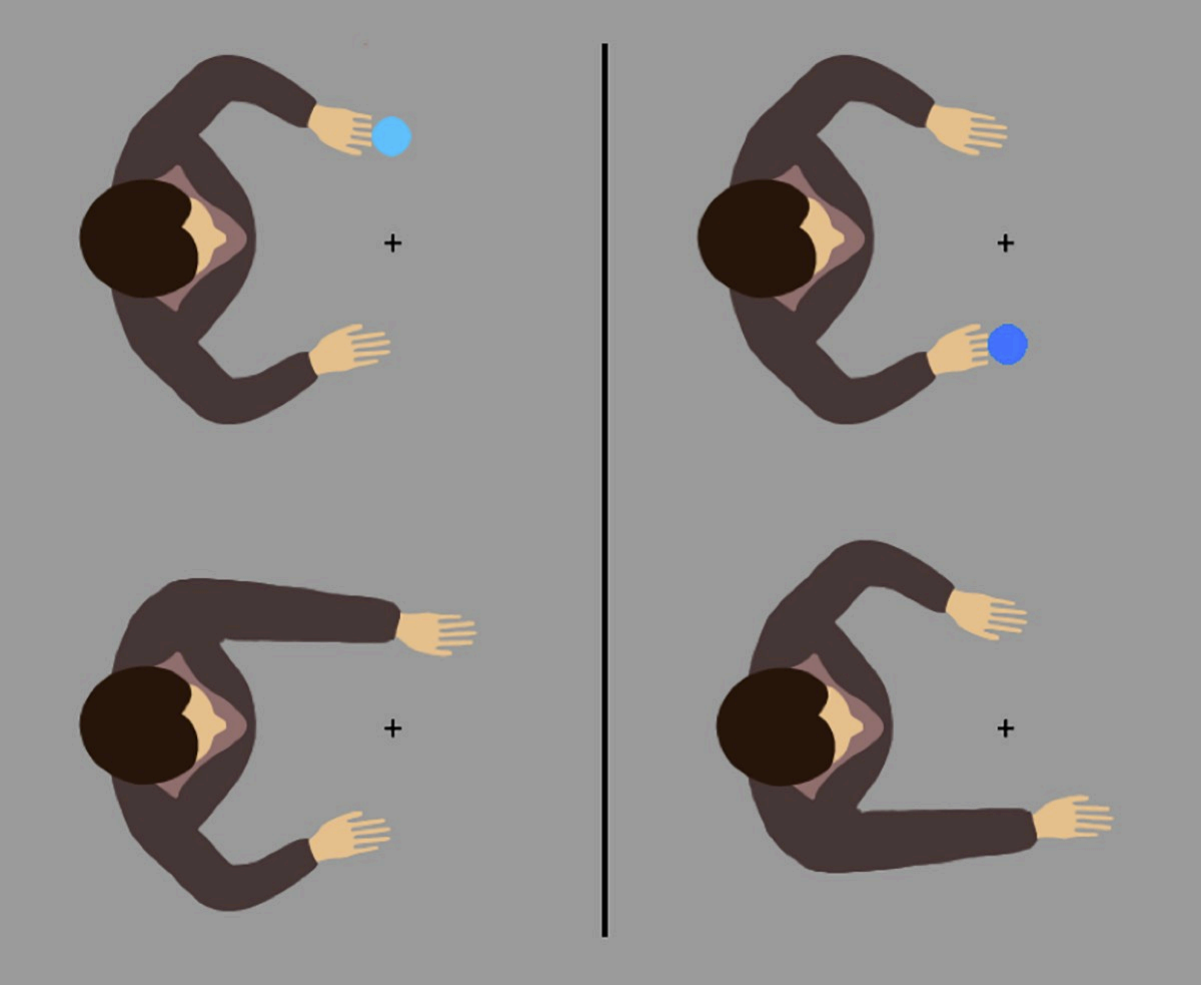
Avatars in Experiment 1. Avatars used in the experiment with the avatar on the left side. The top row shows the initial setup before a response was given, the bottom row illustrates the action effects.

The stimuli were presented on a 22” CRT monitor with a resolution of 1024x768 pixels at a refresh-rate of 100 Hz. Matlab and the Psychtoolbox-3 Extension [29,30] were used to control the experiment. The participants were seated approximately 60 cm in front of the monitor and performed left or right key presses on a horizontally oriented keypad. Stimuli were dark and light blue discs (25 pixels in diameter) presented either above a below a fixation cross and in front of an avatar (240x190 pixels, Fig 1) that was facing the screen center, either from the left or right. The stimuli were presented against a gray background.

#### Procedure

The participants were instructed to perform key presses based on the color of the disc. These discs were presented in the middle of the screen on two vertical positions, equidistant above or below a central fixation cross. Additionally, an avatar was presented on the right or left of the stimulus set, facing the center of the screen and the stimuli. Consequently, the avatar’s hands were pointing towards both stimulus positions (Fig 1 top row). After a block of 192 trials, the avatar switched positions from left to right or from right to left depending on the starting position that was counterbalanced between participants. The discs’ color (light or dark blue) determined the required response. Half the participants were instructed to perform a left key press after a light blue disc, and a right key press after a dark blue disc. The other half used the opposite mapping. After the participants gave their response, the target disappeared and an action effect in the form of an avatar hand movement was displayed, as soon as possible on the next valid frame (Fig 1 bottom row). The delay was kept to a minimum to ensure that response and effect could be integrated into the same event file [14]. In the random condition, the movement was performed with a probability of 50% either with the left or right hand, while in the corresponding condition it was always performed with the corresponding hand of the avatar. The following trial was initiated after a 1500 ms interval. False responses and responses faster than 100 ms or slower than 1500 ms led to an error sound to discourage timeouts and responses based on anticipations. The intertrial interval was prolonged for an additional 1500 ms for each error sound.

The participants performed two sets of blocks of either condition (random or corresponding), each of them with the avatar being on the left or right, followed by two blocks of the other condition. As a result, action effect correspondence switched from random to corresponding after half of the experiment was completed. After each quarter of the experiment, the avatar’s position changed from left to right or vice versa. The avatar’s starting position and the starting action effect correspondence were counterbalanced between participants. All other factors varied on a trial by trial basis and occurred equally often with randomized order in each block. Each block started out with 32 practice trials and was followed by a total of 160 trials with 40 repetitions of each condition. After each half of the experiment, the perceived ownership of the avatar and agency values were estimated using a questionnaire (Table 1). It contained 10 items [23] based on an earlier version of the body ownership questionnaire [31,32] that was adapted to fit the avatar scenario and in which the participants had to state their agreement to 10 items on a 7-point Likert scale. Items 1, 2 and 9 are targeting the ownership illusion and the remaining items can be regarded as filler items [33]. Additionally, one agency item was added as a manipulation check that asked the participants to estimate how often (in %) they felt responsible for the action effect location. The questionnaire was used in a German version and an English translation of the items is shown in Table 1.

**Table 1:** Items used in the ownership questionnaire. The items are based on the instruments developed by Botvinick and Cohen [31] and Ma and Hommel [32] .

O1: It felt as if the avatar’s hands were part of my body.
O2: It seemed that my hand was in the location where the hand of the avatar was.
O3: I lost the feeling where my hands were located.
O4: It seemed that my hands were no longer part of my body.
O5: I had the feeling that I might have additional hands.
O6: Sometimes I felt as if my hands were turning virtual.
O7: The hands of the avatar began to resemble my hands in terms of shape or skin tone.
O8: It appeared (visually) as if the hands of the avatar were drifting towards my hands.
O9: It seemed like I could have moved the hand on the screen if I wanted, as if it were obeying my will.
O10: It felt as if my hands took on the same size as the avatar’s hands.
A: How often (in %) did you feel that your action controlled the location of the hand movement?

#### Design

The avatar’s position, the position of the stimuli and the response positions were collapsed into one factor: *avatar compatibility*. This factor describes stimulus-response compatibility from the avatar’s perspective and key presses were compatible to stimuli presented on the same side, as seen from the avatar’s point of view. The second factor was *action effect* (corresponding or random) resulting in a 2x2 within-subjects design with repeated measures on both factors.

### Results

The first 32 of all blocks were practice trials and not recorded. Outliers (5.5%) were identified using the Tukey criterion (1.5 times the interquartile range above the third or below the first quartile of the reaction time distribution, separately for each person and factor level) and removed from the reaction time analysis. Additionally, false responses (4.3% of all trials) were removed and analyzed separately. We used a 2x2 ANOVA with repeated measures on both factors (*avatar compatibility* and *action effect*) to analyze mean correct reaction times (RT) and percentage errors (PE). We report a η_p_^2^ as an effect size estimate that accounts for the correlation between repeated measures [34].

#### Reaction times

**Fig 2:**
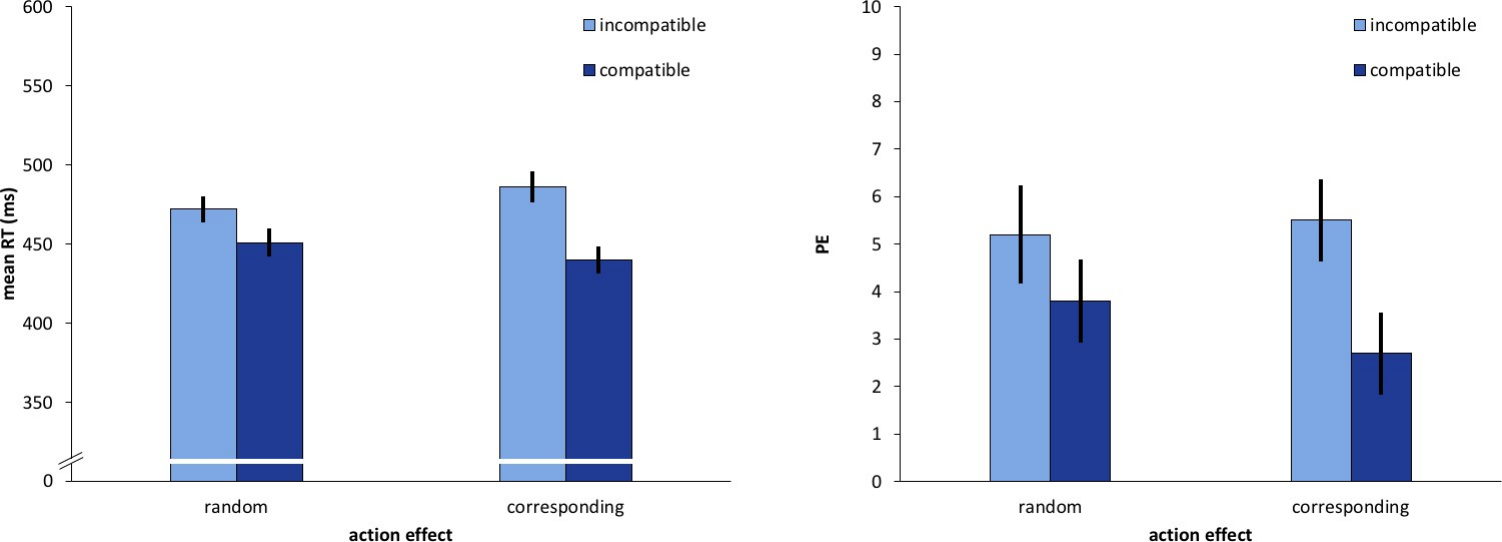
Results of Experiment 1. Mean Reaction times (RT) in ms (left) and percentage errors (PE, right) as a function of avatar compatibility and action effect. Error bars represent 95% within-subject CIs [35].

The results showed a significant main effect of *avatar compatibility*, *F* (1, 23) = 86.60, *p* < .001, η_p_^2^ = .790. Avatar-compatible conditions were associated with a reaction time advantage of 33 ms over avatar-incompatible conditions (Fig 2). We also observed a significant interaction of *avatar compatibility* and *action effect*, *F* (1, 23) = 14.93, *p* = .001, η_p_^2^ = .394. The avatar compatibility effect was more pronounced with corresponding action effects (*M*_corresp._ = 46 ms) compared to random action effects (*M*_rnd._ = 21 ms). Post-hoc two-tailed t-tests showed, that the avatar-Simon effect was significant in both action effect conditions with *t*(23) = 4.97, *p* < 0.01 in the random and *t*(23) = 8.42, *p* < 0.01 in the corresponding action effect condition.

#### Percentage errors

We observed a significant main effect of *avatar compatibility* of *F*(1, 23) = 12.70, *p* = .002, η_p_^2^ = .356 with a 2.1%-points avatar compatibility effect in favor of compatible conditions. Furthermore, *avatar compatibility* and *action effect* interacted significantly with *F* (1, 23) = 5.02, *p* = .035, η_p_^2^ = .179 and the compatibility effect was larger in the corresponding conditions (*M*_corresp._ = 2.8%-points) compared to the random condition (*M*_rnd._ = 1.4%-points). The avatar-Simon effect was significant in the corresponding action effect condition with *t*(23) = 4.43, *p* < 0.01 but not in the random action effect condition *t*(23) = 1.90, *p* = 0.07.

#### Questionnaire data

One answer to the agency question (Table 1: A) was missing and this person was therefore excluded from the analysis of this question. If a person answered the agency question by giving an interval estimate (e.g. “40–50%”), the central value of this interval was used as the answer in the following analysis. To avoid effects of suggestibility, we calculated a corrected ownership score for each participant, by subtracting the mean of the filler items (Q3-8, Q10) from the mean of the ownership items proper (Q1, Q2, Q9), as proposed by [36]. A significantly positive value indicates that ownership is experienced.

The answers of the single agency question and the ownership scores were evaluated separately using one tailed t-tests for repeated measurements, because we predicted higher ownership scores and agency estimates in the corresponding condition. This way, we obtained a corrected ownership score of 0.83, (95% CI [0.49, 1.17]) in the corresponding action effect condition, and a score of 0.35 (95% CI [0.07, 0.64]) in the random action effect condition that are significantly different from one another *t*(23) = 3.17, *p* = .002, one tailed. The participants also reported significantly different values of perceived agency, *t*(22) = 4.41, *p* < .001, one tailed, between both action effect conditions. The reported values were higher with corresponding action effects (*M*_corresp._ = 68.5%) compared to the random action effect condition (*M*_rnd._ = 35.0%).

### Discussion

The results confirm the expected importance of spatially corresponding and therefore predictable and reliable action effects when interacting with an avatar. The participants reported higher perceived agency, which shows, that the action effect manipulation was successful and perceived consciously. The corrected ownership scores confirm that the ownership illusion was present in both action effect conditions and not only a result of suggestibility. Additionally, the participants had an easier time experiencing body ownership of the avatar in the corresponding condition, which makes us believe that corresponding action effects help to destroy the perceptual barrier between oneself and the avatar. A process that can be described as self-other merging [37] and that leads to the person and the avatar forming a functional unit.

A similar result was obtained with regards to the observed avatar-compatibility effects. Even though both, the corresponding and random action effect condition, revealed a compatibility effect from the avatar’s point of view, the effect was significantly larger in the corresponding condition. This experiment therefore provides additional evidence that body ownership and perspective taking of an avatar are related [23].

This leads us to two conclusions: 1. Corresponding action effects aid spontaneous visual perspective taking and 2. random action effects are insufficient when it comes to eliminating the avatar-based compatibility effects. One possible reason for the second observation might be that the hand movement itself is such a natural and therefore easily anticipated effect, that it is not readily disrupted by action-effect inconsistencies. It therefore seems to be a plausible next step to eliminate the anatomical link between avatar and action effect in order to examine, if this leads to an elimination of the action effect influence.

## Experiment 2

The second experiment destroyed the anatomical connection between avatar and action effect by replacing the hand movement with the display of a red disc as an avatar-independent action effect. We expected that this change would disrupt the forming of an action-action effect connection related to the avatar because the effect was no longer linked to the avatar and it turned the task into a basic Simon task with an additional action effect manipulation, that just happened to have an avatar nearby. Even though the participants’ responses were still followed by action effects, they had no control over the avatar itself, because it never moved. In contrast to past studies that used comparable action effect manipulations in a Simon task, e.g. [38], the effect in our study was not defined as the action goal. As a result, the influence of action effect correspondence might vanish as well (compare [39–41]). However, we still expected that stimulus-response compatibility would be determined by the avatar’s position, as is the case in similar tasks without action effects [15].

### Method

**Fig 3:**
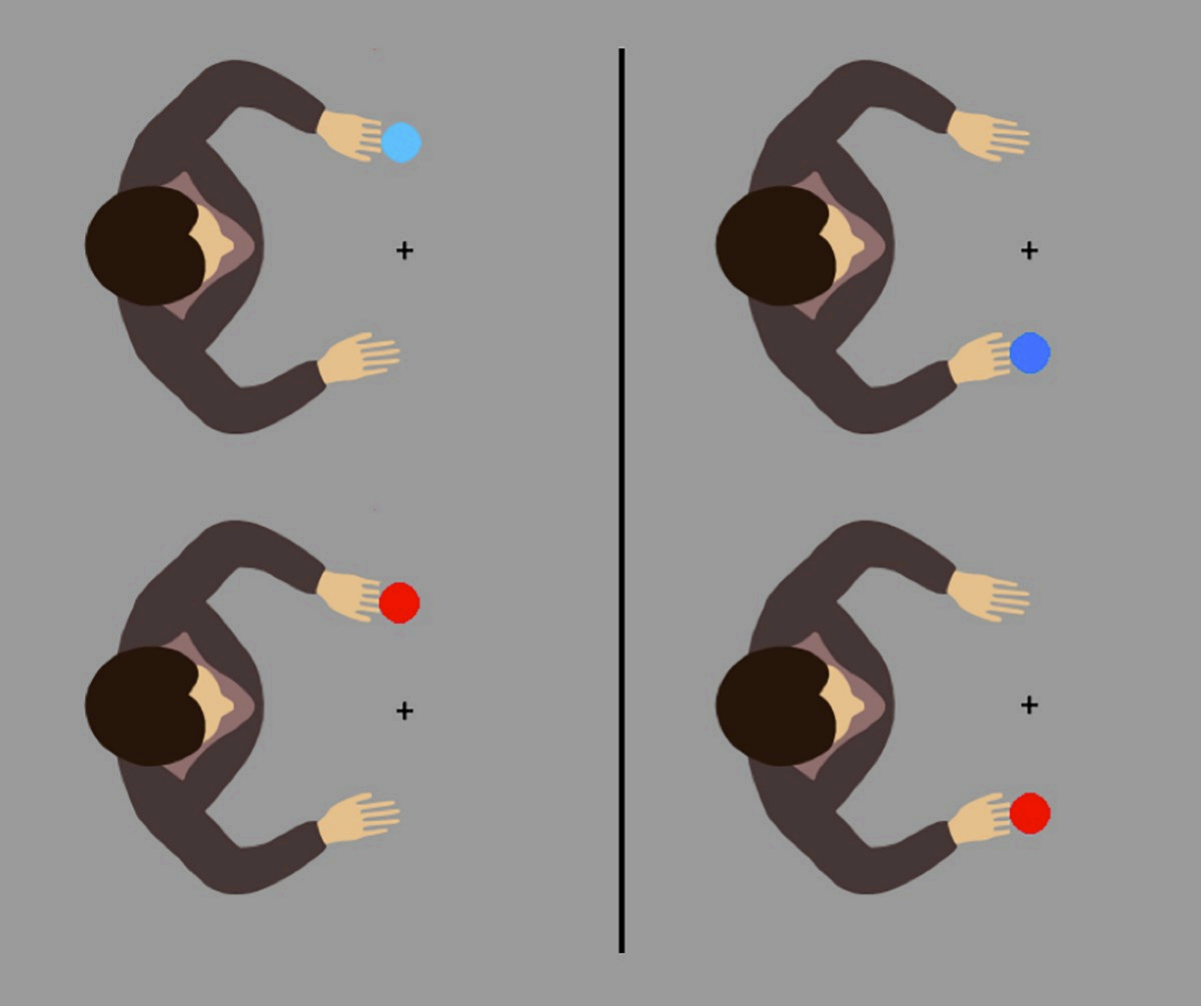
Avatars in Experiment 2. Avatars used in the second experiment with the avatar on the left side. The top row shows the initial setup before a response was given, the bottom row illustrates the two possible action effects.

We used the same procedure as in Experiment 1 but changed the material slightly: Instead of an avatar hand movement, we now used the appearance of a red disc (25 pixels in diameter) as the action effect (Fig 3). We changed the agency question accordingly and asked participants how often (in %) they felt that their response determined the location of the red disc. A total of 24 students (21 female), with a mean age of *M* = 21.5 (*SD* = 3.2) participated in the second experiment.

### Results

We performed the same analysis as in Experiment 1 and excluded 2.8% errors and 4.7% outliers from the RT analysis, based on the same criteria.

#### Reaction times

**Fig 4:**
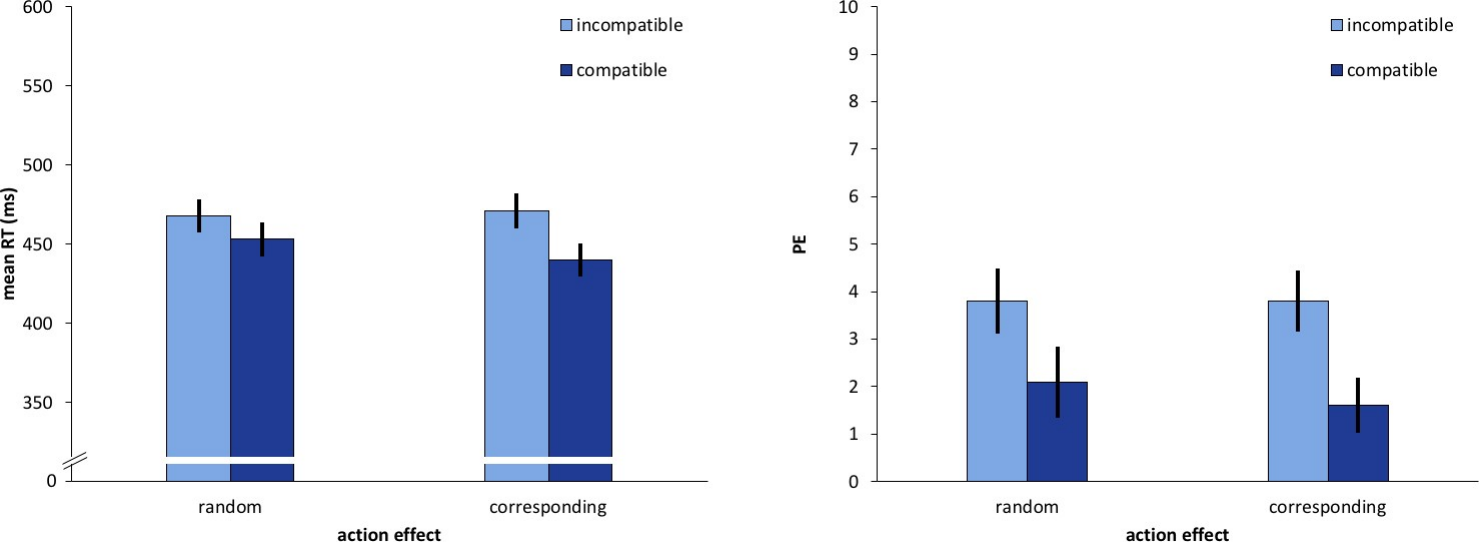
Results of Experiment 2. Mean Reaction times (RT) in ms (left) and percentage errors (PE, right) as a function of avatar compatibility and action effect. Error bars represent 95% within-subject CIs [35].

The results revealed a significant main effect of *avatar compatibility*, *F* (1, 23) = 82.84, *p* < .001, η_p_^2^ = .783. Conditions that were compatible from the avatar’s point of view yielded a reaction time advantage of 23 ms over incompatible ones (Fig 4). We further observed a significant interaction of *avatar compatibility* and *action effect*, *F* (1, 23) = 26.30, *p* < .001, η_p_^2^ = .533. The avatar compatibility effect was again larger with corresponding action effects (*M*_corresp._ = 31 ms) compared to random action effects (*M*_rnd._ = 15 ms). The post-hoc t-tests revealed, that the avatar-Simon effect was significant in both action effect conditions with *t*(23) = 4.94, *p* < 0.01 with random and *t*(23) = 10.79, *p* < 0.01 with corresponding action effects.

#### Percentage errors

The data revealed a significant main effect of *avatar compatibility* with *F* (1, 23) = 19.06, *p* < .001, η_p_^2^ = .453. Avatar-compatible conditions led to 2%-points fewer errors compared to incompatible conditions. A significant interaction effect was not observed in the error percentages. The avatar-Simon effect was significant in both action effects conditions with *t*(23) = 2.95, *p* = 0.07 in the random and *t*(23) = 4.81, *p* < 0.01 in the corresponding action effect condition.

#### Questionnaire data

Two participants did not answer the agency question and were excluded from the analysis of this question. We observed a significant difference in perceived agency between random and corresponding action effects, as reported in the agency item, *t*(21) = 2.02, *p* = .028, (one tailed), with higher values in the corresponding action effect conditions (*M*_corresp._ = 46.1%) compared to the random action effect conditions (*M*_rnd._ = 31.2%). Corrected ownership scores were calculated as in Experiment 1 and we obtained a score of 0.56, (95% CI [0.21, 0.90]) in the random condition and a score of .53 (95% CI [0.20, 0.87]) in the corresponding action effect condition. These corrected ownership scores were not significantly larger in the corresponding condition t(23) = 0.25, p = .403, one tailed.

### Discussion

The results of the second experiment show several parallels to the first. For example, we replicated the avatar-based compatibility effect in both, corresponding and random action effects and again, the avatar-compatibility effect was larger with corresponding in comparison to random action effects. However, the overall avatar-compatibility effects were numerically smaller than in Experiment 1 and an additional analysis is needed to investigate if this difference is statistically relevant (see below). Interestingly, even though the avatar itself did not move in Experiment 2, the unrelated action effect still affected the compatibility effects and therefore spontaneous perspective taking. This means that effects that are unrelated to the avatar can still increase its influence and even coincidental events could potentially influence avatar-based compatibility effects.

Based on the questionnaire data, we can conclude that the action effect manipulation led to different questionnaire results compared to Experiment 1. The corrected ownership scores were significantly positive with either action effect type and did not differ between the two and there is likely no significant difference in perceived ownership of the avatar between corresponding and random action effects. This makes it plausible that the action effect has to be linked to the avatar to be relevant when determining perceived body ownership of the avatar. If the action effect is independent and therefore not directly linked to the avatar, it seems only logical that it is perceived differently. But it is important to note, that the results still indicate that the action effect is regarded within the reference frame provided by the avatar, or else we would not have been able to observe the interaction between *avatar-compatibility* and *action effect*.

Overall, we observed a disparity between two measures of a person’s identification with an avatar. Objective measures of spontaneous perspective taking were influenced by the type of action effects, even when they were not linked to the avatar itself. The participants showed greater compatibility effects from the avatar’s perspective with corresponding action effects compared to random action effects, indicating improved perspective taking. However, the subjective measures of body ownership revealed that this action effect manipulation did not alter the participants’ subjective experience, even though the answers to the agency question showed that the manipulation itself was noticed. While it has been shown in the past that body ownership and perspective taking of an avatar are related [23], the results of the present study show that they can be differently influenced by the same manipulation. It therefore seems likely that a successful manipulation of body ownership might cause an increase in spontaneous perspective taking, however, this is probably not a necessary condition, because differences in perspective taking can be achieved by manipulations that do not influence perceived body ownership.

### Comparing Experiment 1 and 2

To examine whether the differences between both experiments are statistically significant, a combined ANOVA with the additional factor *experiment* was conducted. The results of the RT analysis revealed that the avatar compatibility effect was larger in the first experiment (*M*_E1_ = 33 ms) with anatomically linked action effects compared to the second experiment (*M*_E2_ = 23 ms) with independent action effects, resulting in a significant two-way interaction of *experiment* and *avatar compatibility* with *F* (1, 46) = 4.774, *p* = .034, η_p_^2^ = .094. However, the avatar compatibility effect was not differently influenced by the action effect correspondence between both experiments and the related three-way interaction was not significant *F* (1, 46) = 1.54 *p* = .221, η_p_^2^ = .032.

The analysis of percentage errors did not reveal any significant influence of the factor *experiment*.

The analysis of the questionnaire data revealed that the two experiments did not significantly differ in overall agency ratings: *F* (1, 43) = 2.84 *p* = .099, η_p_^2^ = .062. Furthermore, the difference between random and corresponding action effects in perceived agency was comparable in both experiments with an interaction of *experiment* and *action effect* of *F* (1, 43) = 3.09, *p* = .086, η_p_^2^ = .067.

**Fig 5:**
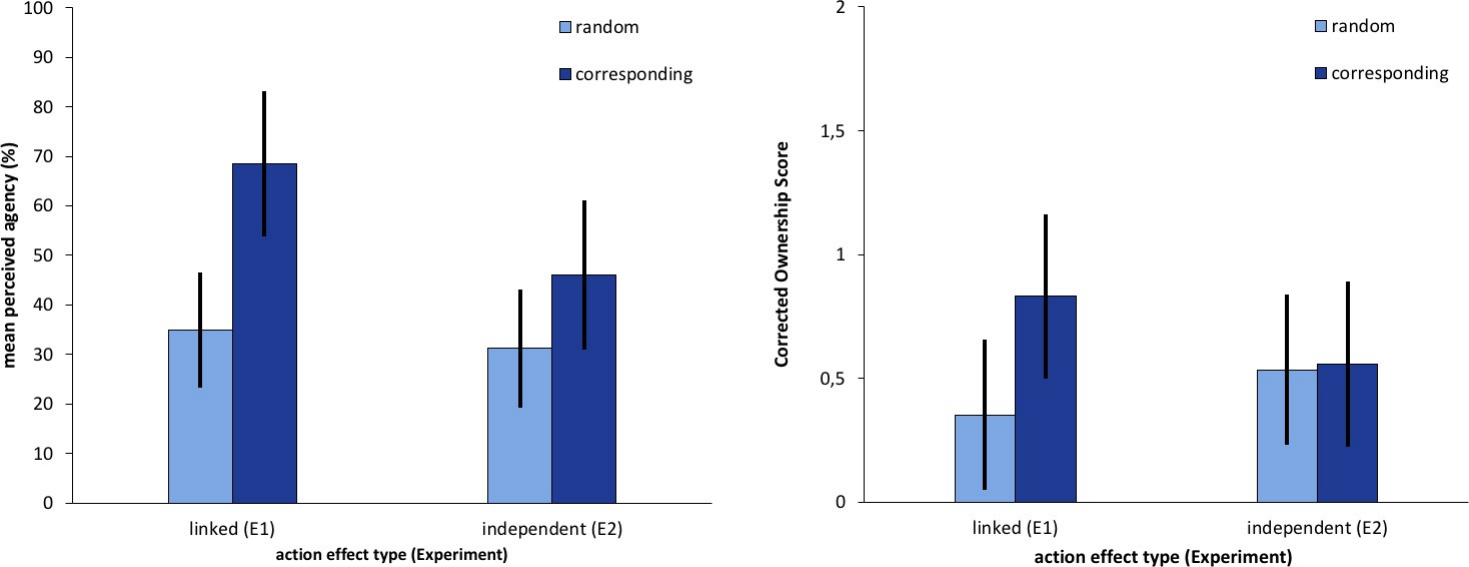
Mean reported agency and corrected ownership scores of Experiment 1 (E1) and 2 (E2). Error bars represent 95% between-subject CIs.

However, while both experiments did result almost identical perceived ownership overall, *F* (1, 46) = .05, *p* = .82, η_p_^2^ = .001, the interaction effect of *experiment* and *action effect* was significant with *F* (1, 46) = 6.47, *p* = .014, η_p_^2^ = .123 (Fig 5) and revealed larger differences between random and corresponding action effects in Experiment 1 compared to Experiment 2.

## General discussion

The results of both experiments allow us to draw several conclusions: First, it has proven useful to implement corresponding action effects if the goal is to facilitate spontaneous visual perspective taking of the avatar. With corresponding action effects, the avatar can be seen as a tool that transforms a key press (proximal effect) into a distal effect (changes on the screen) and this transformation might follow similar rules as the use of simpler tools [42]. This seems to be an overarching phenomenon as it is not limited to anatomically linked action effects and can instead also be observed for independent action effects. If we look at the compatibility differences between both experiments, the effects are overall larger in the anatomically linked action effect scenario of Experiment 1. So, if we want to maximize spontaneous perspective taking, we should use a setup with reliable and linked action effects. But if our design space is limited to non-embodied action effects, we should also pay attention to action effect correspondence in order to maximize perspective taking, a result that seems surprising from the standpoint of embodiment. We can also think of several cases where a different design goal might be more important. For example, in an application that uses virtual agents in a teaching context or in a navigation tool, it could prove detrimental if the users spontaneously take this avatar’s perspective over their own. In this case, actively avoiding any effect that could be interpreted as an action effect might be beneficial, especially, if the action effect is performed by the agent and always corresponds to the user’s input.

However, the results of the subjective measurements might indicate that the situation is different when it comes to perceived body ownership of the avatar. Here, the embodied nature of the action effect likely comes into play. While corresponding and anatomically linked action effects do produce the highest reported body ownership values, the case of independent action effects is not as clear-cut. In an independent action effect scenario, action effect correspondence seems to be of secondary importance as it does not seem to significantly increase perceived ownership compared to the random condition. When contrasting the ownership values of both experiments, the experiment—action effect interaction did not reach significance, even though it came very close. One possible reason for this is, that the subjective nature of the measurement is less reliable compared to the objective reaction time measurements. This is enhanced by the fact that the comparisons within each experiment were within-subject comparisons, while the comparisons between the experiments are influenced by between-subjects variance. It is also possible that there are big interindividual differences when it comes to the interpretation of the action effects. It has been shown in the past that people can be categorized as either “embodiers” or “systemizers” when they complete similar tasks [43]. “Embodiers” usually show strong embodiment effects that are reduced or absent in “systemizers”. It is possible that similar effects are at work in the current study, and that some people have an easier time experiencing an action effect as linked to the avatar or themselves compared to others.

Overall this points us to the conclusion that our perception of the avatar and the observed compatibility effects from its point of view can be disjointed in certain set-ups. Whereas in more natural settings that use linked and therefore easily anticipated action effects, different mechanisms seem to be relevant when it comes to the perception of independent action effects. Whether these effects are social in nature is still unclear. However, the conditions with reliable and corresponding action effects share a lot of characteristics with tool-use scenarios. In this case, the avatars themselves are not social agents but means of interaction with a virtual environment. Additionally, if we expect that avatars can be fully integrated into a person’s self-representation, then the social aspect is again lost, because the avatar and the person form a functional unit. With this in mind, theoretical frameworks that do not rely on the social aspect of the human-avatar interaction—such as referential coding [19] or a broad definition of perspective taking [20]—appear to suited best in order to explain the underlying mechanisms of avatar-based compatibility effects.

## Conclusion

The goal of this experiment was to examine the role of action effects for spontaneous perspective taking and perceived body ownership of task-irrelevant avatars. While past studies were able to demonstrate a relationship between both concepts using a top-down approach [23], this connection was only found in a bottom-up approach here, when the action effect was linked to the avatar. Even though we observed differences in avatar-compatibility between both experiments, the fundamental mechanism seems to hold true in both cases: The spatial coding of the scene seems to always occur with regards to the reference frame provided by the avatar and we can use stimulus-response compatibility to objectively measure these effects. In contrast to this, the subjective experience appears to rely more heavily on the nature of the action effect. Both questions regarding the action effect examined in this study seem to matter for perceived body ownership of the avatar: 1. Is the effect linked to the avatar? and 2. Is the effect predictable and reliable? This leads us to an interesting distinction between objective and subjective measures of the human-avatar interaction and different approaches should be used depending on the features of the scenario in question and the limitations of the design space in order to maximize the identification with the avatar or to prevent it.

## Supporting information

S1 File Data. This dataset contains mean reaction times, error rates and questionnaire results per condition and participant.
(CSV)

S2 File Readme. This file contains brief explanations of the variables reported in the dataset.
(TXT)
